# Essential validation methods for *E. coli* strains created by chromosome engineering

**DOI:** 10.1186/s13036-015-0008-x

**Published:** 2015-07-01

**Authors:** Sriram Tiruvadi Krishnan, M. Charl Moolman, Theo van Laar, Anne S. Meyer, Nynke H. Dekker

**Affiliations:** Department of Bionanoscience, Kavli Institute of Nanoscience, Faculty of Applied Sciences, Delft University of Technology, Lorentzweg 1, Delft, 2628 CJ The Netherlands

**Keywords:** Chromosome engineering, *Escherichia coli*, Recombineering, P1 phage transduction, Strain validation, EBU plate assay, Growth curve analysis, Cell shape analysis

## Abstract

**Background:**

Chromosome engineering encompasses a collection of homologous recombination-based techniques that are employed to modify the genome of a model organism in a controlled fashion. Such techniques are widely used in both fundamental and industrial research to introduce multiple insertions in the same *Escherichia coli* strain. To date, λ-Red recombination (also known as recombineering) and P1 phage transduction are the most successfully implemented chromosome engineering techniques in *E. coli*. However, due to errors that can occur during the strain creation process, reliable validation methods are essential upon alteration of a strain’s chromosome.

**Results and discussion:**

Polymerase chain reaction (PCR)-based methods and DNA sequence analysis are rapid and powerful methods to verify successful integration of DNA sequences into a chromosome. Even though these verification methods are necessary, they may not be sufficient in detecting all errors, imposing the requirement of additional validation methods. For example, as extraneous insertions may occur during recombineering, we highlight the use of Southern blotting to detect their presence. These unwanted mutations can be removed via transducing the region of interest into the wild type chromosome using P1 phages. However, in doing so one must verify that both the P1 lysate and the strains utilized are free from contamination with temperate phages, as these can lysogenize inside a cell as a large plasmid. Thus, we illustrate various methods to probe for temperate phage contamination, including cross-streak agar and Evans Blue-Uranine (EBU) plate assays, whereby the latter is a newly reported technique for this purpose in *E. coli*. Lastly, we discuss methodologies for detecting defects in cell growth and shape characteristics, which should be employed as an additional check.

**Conclusion:**

The simple, yet crucial validation techniques discussed here can be used to reliably verify any chromosomally engineered *E. coli* strains for errors such as non-specific insertions in the chromosome, temperate phage contamination, and defects in growth and cell shape. While techniques such as PCR and DNA sequence verification should standardly be performed, we illustrate the necessity of performing these additional assays. The discussed techniques are highly generic and can be easily applied to any type of chromosome engineering.

## Background

Modification of an organism’s phenotypes by altering its chromosomal DNA sequence in a controlled manner provides the fundamental motivation for chromosome engineering [1]. This engineering comprises a collection of techniques that can be applied to insert foreign DNA sequences at a specific locus, delete the native sequence, or alter the bases in the chromosomal DNA of a model organism. One such key model organism is the bacterium *Escherichia coli* (*E. coli*), used for many fundamental studies in molecular and cell biology, and it is also utilized for expressing novel proteins. The single, circular chromosome of *E. coli* has been fully sequenced and amply annotated, paving the way for researchers to precisely engineer its chromosome using a variety of methods [2]. Most chromosome engineering techniques in *E. coli* harness the properties of the recombinase family of enzymes, expressed by plasmids or bacteriophages, which recombine homologous linear DNA fragments into the host chromosome [3].

In the past decade, numerous studies have successfully employed chromosome engineering tools in *E. coli* research. The ability to fuse a fluorescent protein gene with native genes [4] is an example that has opened up the possibility of live cell imaging to visualize the dynamics and stoichiometry of native proteins involved in key biochemical processes such as DNA replication [5, 6], transcription [7, 8], translation [7], chromosome segregation [9], cell signaling [10], and flagellar motor dynamics [11]. Similarly, a high throughput study in which single genes were systematically deleted from the *E. coli* chromosome has shed light on the (non)-essential character of individual genes, and hence on the possibilities of creating a minimal cell containing only the most essential genes [12, 13]. In addition to such studies into fundamental aspects of molecular and cellular biology, chromosome engineering is also used in industrial research to produce essential bio-chemicals, bio-fuels, and precursors for pharmaceuticals on a large scale by engineering all necessary genes into a single *E. coli* strain [14–16]. In industrial research, chromosome engineering is typically preferred to conventional cloning in plasmids, as it obviates the need for antibiotics to maintain gene presence [15].

For any research in which the chromosome is engineered, it is essential to reliably verify that the process has not inadvertently introduced anomalies into the genome. For example, the use of λ-Red recombination or recombineering [17–20] (Table 1) may result in insertions at undesired locations in the chromosome, a result of sequence heterogeneities introduced during synthesis of the requisite long primers [17, 21], the presence of an unstable genomic region [22], or the occurrence of partial gene duplication in the chromosome during the strain creation process [23]. The latter is illustrated by the gene duplication errors that occurred in 0.6 % of the 3864 single-gene deletion mutants of *E. coli* K12 strains in the Keio collection [23]. Together with the intended insertion which usually occurs at a probability of ~10^−4^ to 10^−5^ [24] non-specific mutations may occur and, they are not detected easily using standard PCR techniques [22]. When such errors occur, P1 phage transduction (Table 1) can be performed to recover the strain of interest, as the region of interest can be specifically transduced into a clean wild type strain following recombineering [25–31]. However, P1 phage transduction comes with its own challenges, such as the potential contamination of temperate phages in the phage stock that can lysogenize as a large plasmid in the created strain. Hence, appropriate validation of the chromosomally engineered strain remains a critical step in the strain creation process.

**Table 1:** Chromosome engineering techniques widely used in *E.coli*

λ-Red recombination
The λ-Red recombination (or recombineering) approach has been successfully implemented in many studies to engineer specific sites in the *E. coli* chromosome [17]. In this approach, chromosomal sequences are replaced by a linear DNA fragment (flanked with sequences homologous to the region of interest) through the use of a temperature-sensitive plasmid that expresses either the Red recombinase genes (*bet*, *gam* and *exo*) from λ-phage [17] or the RecET proteins from Rac prophage [18] upon induction. The linear DNA fragment of interest is usually synthesized via PCR, in which case the homologous sequences (~50 bases) are introduced through the employed primers. The recombined strain is selected using a constitutively expressed antibiotic marker that is integrated into the chromosome along with the insert of interest. This technique may also be combined with FLP/*FRT-*based recombination, in which the antibiotic marker is flanked by *FRT* sites that allow it to be recombined out using the flippase (FLP) enzyme [19]. In this way, the created strain may be employed in multiple rounds of chromosome engineering using the same antibiotic marker [17, 20].
Generalized P1 phage transduction
Generalized P1 phage transduction is widely used to transfer mutations from one *E. coli* strain to another with the same genetic background [26, 27]. This approach is based on the fact that virulent P1 phages commit errors while packaging their DNA into coat proteins: instead of packaging their own genome, they package lysed host chromosomal DNA fragments [28–30]. Such mis-packaged phages form approximately ~5 % of the total phage population in a lysate. When they are transduced into a different host, the chromosome fragment may be inserted precisely at a homologous site using the RecA-dependent system [26]. Using this approach, multiple insertions can be made into the ~4.6 Mb chromosome of same *E. coli* strain, provided that they are separated by ~100 kb [31]. A combination of λ-Red recombination, FLP/FRT recombination, and P1 phage transduction methods can also be used to introduce multiple insertions into a single *E. coli* strain [6, 15].

Here, we describe in detail a number of general methods for the validation of strains with altered chromosomes, and accompany this description with experimental results. We note that several of the techniques described here are individually well known to the scientific community; however, frequently only the more standard verification procedures for chromosome engineering, PCR and DNA sequence analysis, are typically reported. Grouping together the description of these techniques, we bring to the increased attention of researchers the most common defects that can arise during strain creation, together with the appropriate methods to verify them. In doing so, we hope to make these techniques more readily accessible to a wider community, facilitating access to them by new researchers and/or those engaged in cross-disciplinary study. We have organized our description of these essential validation methods along the lines of the irregularities that may occur: (i) non-specific insertions in the chromosome; (ii) the contamination of temperate P1 phage in the engineered *E. coli* strains; and (iii) defects in phenotypes such as cell growth and morphology. The latter physiological aspect must be taken into account if the results obtained from an engineered *E. coli* strain are to be generalized to wild type *E. coli*. Within each of these categories, we illustrate the defects that can arise from errors in chromosome engineering and describe various methods to detect them, using as examples the creation of two *E.coli* AB1157 strains: non-motile *ΔmotAB* for use in live-cell fluorescence microscopy [32] and *pBad-DnaG* in which an inducible primase gene is inserted into the non-essential *galK* chromosomal locus.

## Results and discussion

### Verification of an engineered sequence in the chromosome

**Fig. 1:**
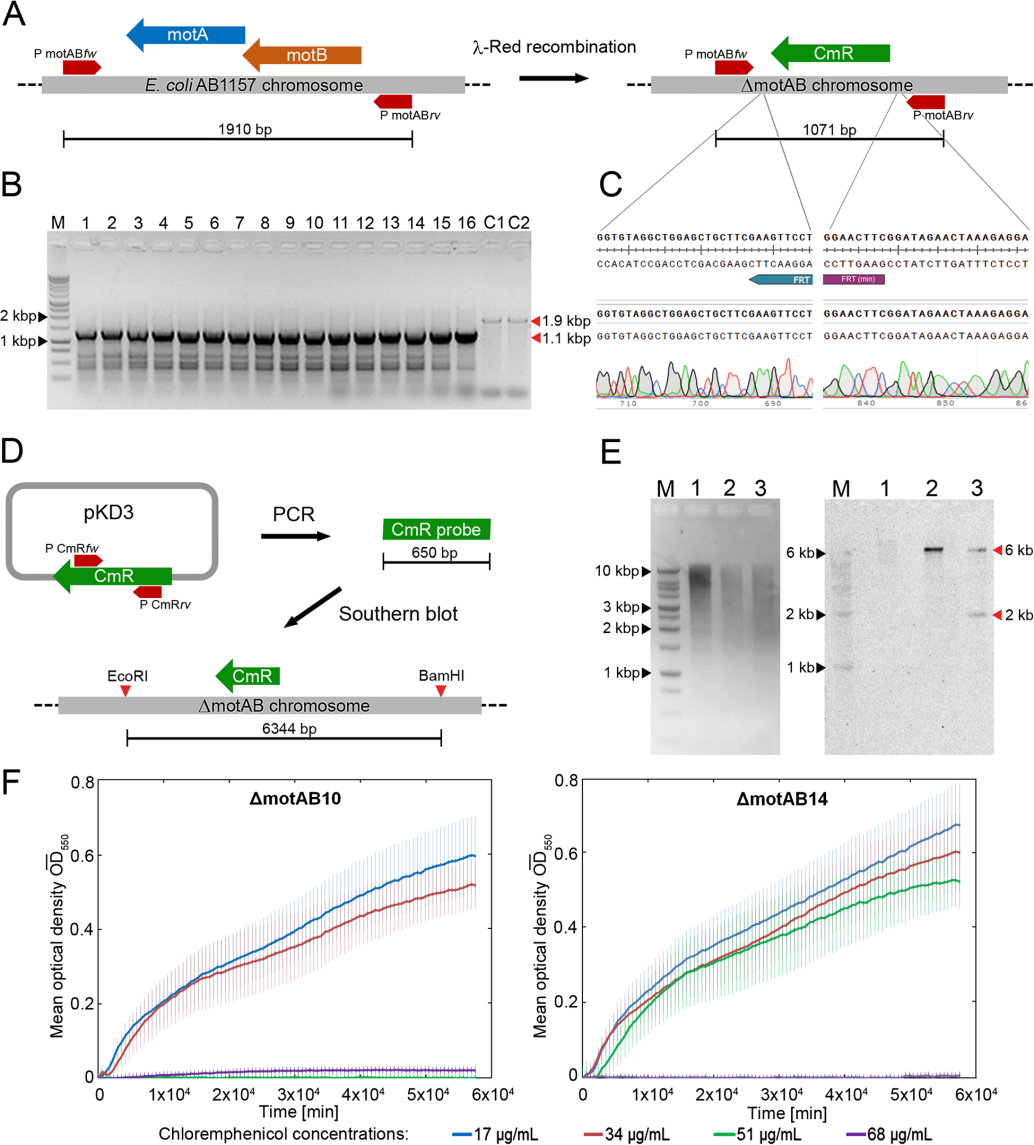
Verification of an engineered sequence in the chromosome. **a** The scheme depicts the changes at the recombineering site to create the *motAB* gene knockout strain (*ΔmotAB*) using a chloramphenicol resistance gene (*CmR*). The positions of the flanking primers for the motAB region (PmotAB*fw* and PmotAB*rv*) are marked, and the corresponding product lengths from PCR are indicated at the bottom. **b** PCR results of the colonies obtained through recombineering. In lane M, 10 μL of DNA ladder was loaded. In lanes 1–16, 10 μL of PCR products from selected, individual *ΔmotAB* colonies were loaded (*ΔmotAB*1 to *ΔmotAB*16). The PCR products of the control AB1157 strain were added in wells C1 and C2. PCR products of the intended sizes are visible for all 16 selected colonies (~1.1 kbp marked with red arrow) as well as the AB1157 colonies (~1.9 kbp). **c** A representative DNA sequencing result of the *ΔmotAB*10 strain at the sites of integration and the corresponding alignment with the expected template DNA sequence are shown. **d** The important steps of making the Southern blot probes are illustrated. A 650 bp PCR product is amplified from the template plasmid pKD3 and is then labelled with alkaline phosphatase to probe the *CmR* region (expected size: 6 kbp). **e** The ethidium bromide stained gel containing the DNA ladder (lane M), the restriction-digested AB1157 genome (lane 1), restriction-digested genomes of two *ΔmotAB* colonies (lane 2: *ΔmotAB*10 and lane 3: *ΔmotAB*14 which were verified by PCR and DNA sequencing). The Southern blot results show that the AB1157 sample in lane 1 has no insert, as expected; lane 2 with *ΔmotAB*10 has one band (6 kbp) at the right fragment size showing that the integration was successful at the predicted site; lane 3 with *ΔmotAB*14 has two bands (6 kbp and 2 kbp). **f** The growth of *ΔmotAB*10 and *ΔmotAB*14 strains in 96 well-plate reader containing LB medium with various concentrations of chloramphenicol (17 μg/mL to 68 μg/mL). The results show that *ΔmotAB*14 strain containing the extraneous insertion grew at a higher concentration of chloramphenicol (51 μg/mL) than the normal concentration (34 μg/mL), while the *ΔmotAB*10 did not grow at 51 μg/mL of chloramphenicol

PCR and DNA sequence analysis are the techniques that are widely performed and reported to verify whether a chromosome engineering technique has successfully modified the chromosomal DNA sequence. We performed these well-known techniques as a first pass in the validation process of strains created via λ-Red recombination. Specifically, we performed λ-Red recombination to knock out the genes expressing the flagellar motor proteins (*ΔmotAB*) in the *E. coli* AB1157 strain (Methods section IA). The targeted gene was replaced with the chloramphenicol resistance gene (*CmR*), which was used as a selection marker to isolate the successfully engineered colonies. To verify whether the insert (*FRT*-*CmR*-*FRT*) was located at the intended site, we designed primers that flank the region of interest (Fig. 1a, Table 2). The positive results of a PCR reaction performed on the 16 selected colonies using flanking primers indicated that the *motAB* genes in the chromosome were replaced with the *CmR* gene (Fig. 1b). DNA sequence analysis was performed to verify the recombineered region of the chromosome (Methods section IIA). The quality of sequencing results also provides insights on the integrated DNA sequence in chromosome. For example, if the DNA sequence results show a double signal (i.e. signals for two bases at the same position), it indicates sequence heterogeneity of the integrated DNA amongst the cells of a colony [33]. In our experiments, the DNA sequence and its alignment with the template sequence revealed that the *motAB* genes were successfully knocked out without sequence errors in 9 out of 16 colonies (Table 3). A representative DNA sequencing result of the *ΔmotAB*10 strain at the sites of integration and the corresponding alignment with the expected template DNA sequence are shown (Fig. 1c).

**Table 2:** Primer names and sequences used

Primer name	Primer sequence
PmotAB*fw*	5′- GCTGAAGCCAAAAGTTCCTG-3′
PmotAB*rv*	5′- TGCCTGCAGCTTATGTCAAC-3′
PcmR*fw*	5′- ATCACAAACGGCATGATGAA-3′
PcmR*rv*	5′- TCACTACCGGGCGTATTTTT-3′
PgalK*fw*	5′- TCCATCAGCGTGACTACCATC-3′
PgalK*rv*	5′- CAGAACAGGCAGCAGAGCGT-3′

**Table 3:** Summary of DNA sequence analysis results for various *ΔmotAB* colonies

Colony id	Summary of DNA sequence analysis results
*ΔmotAB*1	Positive (Good signal at both the integration sites and insert)
*ΔmotAB*2	Positive (Good signal at both the integration sites and insert)
*ΔmotAB*3	Negative (Double signal at the end)
*ΔmotAB*4	Positive (Good signal at both the integration sites and insert)
*ΔmotAB*5	Positive (Good signal at both the integration sites and insert)
*ΔmotAB*6	Negative (Low signal)
*ΔmotAB*7	Negative (Double signal at the end)
*ΔmotAB*8	Negative (Double signal at the beginning)
*ΔmotAB*9	Negative (Low signal)
*ΔmotAB*10	Positive (Good signal at both the integration sites and insert)
*ΔmotAB*11	Positive (Good signal at both the integration sites and insert)
*ΔmotAB*12	Negative (Low signal with broad peaks)
*ΔmotAB*13	Negative (Low signal)
*ΔmotAB*14	Positive (Good signal at both the integration sites and insert)
*ΔmotAB*15	Positive (Good signal at both the integration sites and insert)
*ΔmotAB*16	Positive (Good signal at both the integration sites and insert)

Multiple copies of the insert sequence (*CmR*) could have recombined elsewhere in addition to the intended site on the chromosome, and such extraneous insertions can be detected using Southern blotting (Methods section IIB) [22, 24, 34]. We performed this technique on nine *ΔmotAB* strains and AB1157 strain as a negative control, all of which were initially verified by PCR and DNA sequence analysis. For this specific experiment, the chromosomal DNA samples isolated from the strains were first digested with two high fidelity restriction enzymes: EcoRI-HF and BamHI-HF, selected based on the criteria detailed in Methods section IIB. The digested DNA fragments were separated via agarose gel electrophoresis and were then transferred to a blotting membrane to probe for the *CmR* gene. The DNA sequence that serves as a probe was obtained by PCR-based amplification of a 650 bp fragment obtained from the *CmR* gene of template plasmid pKD3 (Table 2, Fig. 1d). This single-stranded probe DNA was labelled directly with thermo-stable alkaline phosphatase enzyme. Following hybridization on the blotting membrane, this enzyme catalyzes a chemi-luminescence reaction, thereby allowing target DNA fragments complementary to the probe to be detected on a CCD detector. Using this approach, we observed that in two of the nine strains (*ΔmotAB*11 and *ΔmotAB*14) the *CmR* gene had recombined not only at the intended region, but also at another non-essential unknown region of the chromosome.

A comparison of the blotting results for *ΔmotAB*10 strain with single intended *CmR* insertion and *ΔmotAB*14 strain with extraneous insertion is shown (Fig. 1e). We also verified that the *ΔmotAB*14 strain showed increased chloramphenicol tolerance compared to the *ΔmotAB*10 strain as a result of this additional insertion. To do so, both strains were grown under constant shaking at 37 °C and 350 rpm in 96 well plates containing LB media with different concentrations of chloramphenicol, and the optical density was measured at regular intervals. While no growth was observed for the *ΔmotAB*10 strain in LB medium including an increased concentration of chloramphenicol (51 μg/mL compared to a normal dosage of 34 μg/mL), the growth of *ΔmotAB*14 strain remained unaffected (Fig. 1f). These results demonstrate that extraneous mutations may occur when performing recombineering techniques [24], supporting the need for strain verification steps like Southern blot analysis in addition to the standard methods of PCR and DNA sequence analysis. The properly verified *ΔmotAB*10 strain generated in these experiments will be referred as *ΔmotAB* in what follows.

### Detection of temperate phage contamination in a phage lysate or a transduced strain

In a phage-transduced strain, temperate P1 bacteriophage can lysogenize as a large plasmid and can replicate for generations along with chromosome of the strain. To demonstrate detection techniques for this phenomenon, we first performed a sample P1 phage transduction experiment. In this experiment, we employed a donor strain in which the β-clamp gene (*dnaN*) is fused with the gene for a yellow fluorescent protein adjacent to a kanamycin marker (*kanR*), as reported in a recent study on DNA replication (*Ypet-DnaN*) [5]. Transduction of such a DNA sequence into the non-motile *ΔmotAB* strain can provide a general approach for live cell imaging studies, whose focus on the visualization of internal cellular dynamics benefits from the use of immobilized cells [32]. As a proof-of-principle for our validation techniques, however, we employed *E. coli* AB1157 strain as the recipient strain.

**Fig. 2:**
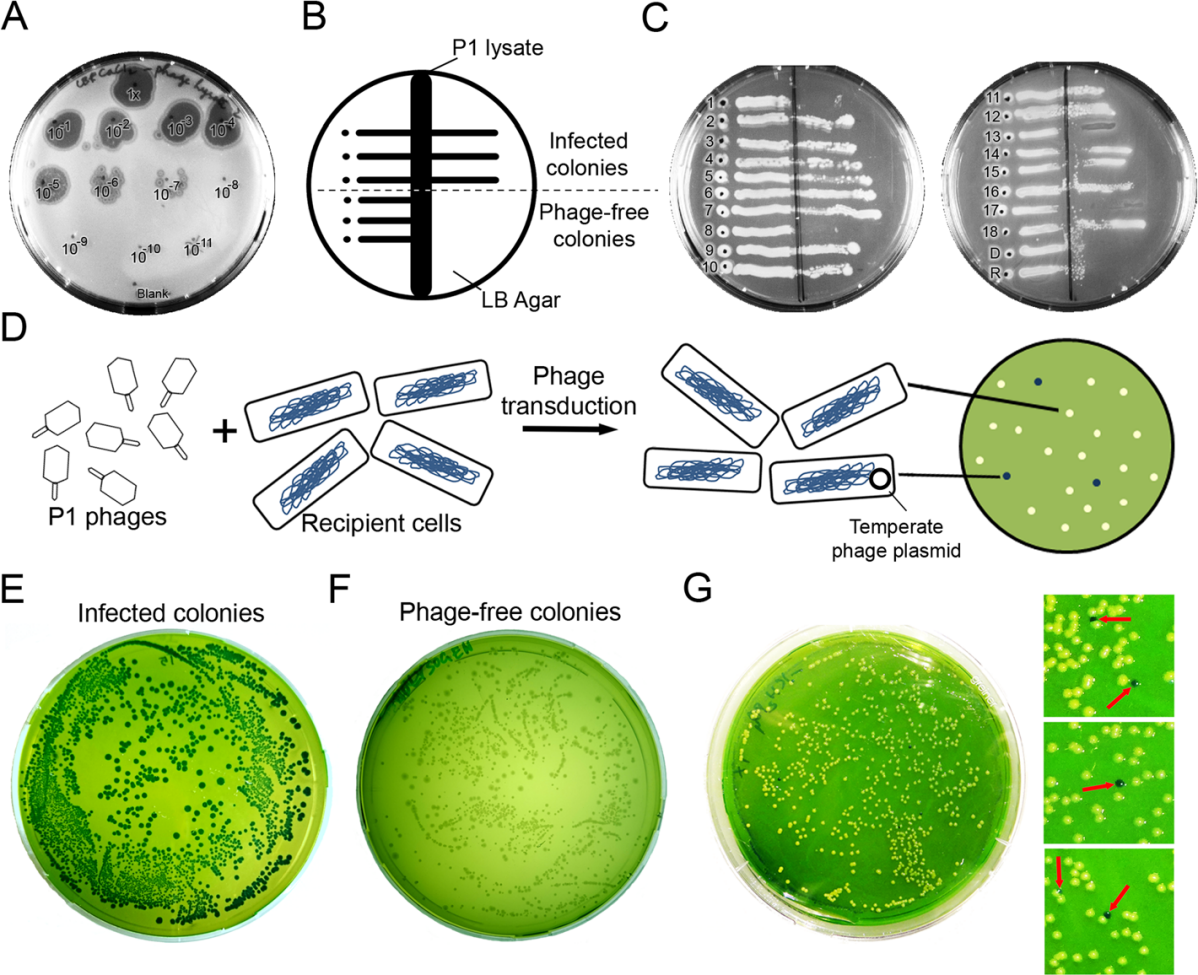
Detection of temperate phage contamination in a phage lysate or transduced strain. **a** The spot agar assay was performed using a serially diluted P1 phage lysate starting from the un-diluted lysate (labelled as 1×) to the dilution factor of 10^11^ (labelled as 10^−11^). The results reveal that plaques are observed at every concentration down to the 10^7th^ dilution. The P1 lysate stock was thereby determined to have an infection titer value of 7 × 10^9^ pfu mL^−1^. **b** A schematic diagram of a typical LB agar plate used for the cross-streak agar assay: The vertical dark region at the center represents the zone of P1 lysate. The dot represents the location where the tested cells are inoculated at a safe distance from lysate zone, and the horizontal solid lines represent either the temperate phage-infected cells that are growing across the lysate zone or the phage-free cells that are not growing beyond the lysate zone. **c** The two representative plates of the cross-streak agar assay performed with the colonies obtained from phage transduction experiment demonstrate that 14 of the 18 colonies tested are infected with temperate phages. The plate on the right side indicates that the donor (D) and recipient (R) strains used are devoid of phages. **d** A scheme of the steps involved in Evans Blue-Uranine (EBU) plate assay, explaining the principle of this technique to screen for temperate phage contamination. **e** Temperate phage-containing cells verified using cross-streak agar assay grew as dark green colonies on EBU plate. **f** Cells verified to be free of phages grew as pale green colonies on EBU plate. **g** An EBU plate assay was performed with the diluted cultures of colonies obtained from a P1 phage transduction experiment. A representative result plate and an enlarged view of the colonies obtained from various EBU plates are shown. A mix of uninfected colonies (pale yellowish green color) containing no temperate phages and colonies containing temperate phages (dark green) were observed on the plates

Prior to phage transduction in *E. coli,* P1 lysate stock should be tested to determine both the infectivity of the P1 phages in the stock as well as the sensitivity of the *E. coli* strain used for transduction. To visualize plaque formation and determine the infection titer value of the P1 stock, we performed a spot agar assay [25, 31]. In this assay, different dilutions of P1 lysate stock are spotted onto a lawn of cells grown on a soft LB agar (0.75 %). The titer values are determined in terms of plaque forming units per mL (pfu mL^−1^). We performed a spot agar assay on the *E. coli* AB1157 strain (Methods section IIC) and, by counting the plaques formed during the assay, determined the titer of the tested P1 lysate to be ~7 × 10^9^ pfu mL^−1^ (Fig. 2a). The observed titer value was found to be in the optimal range for successful transduction (10^9^ to 10^10^ pfu mL^−1^) [25]. Phage transduction was performed using the characterized lysate on *Ypet-DnaN* as the donor strain and AB1157 as the recipient strain (Methods section IB).

One must carefully ensure that phage-transduced cells do not harbor temperate phages, which can result for instance from the use of a P1 lysate contaminated with temperate phages. This phenomenon can yield undesirable results, such as slow growth or abnormal physiology, in the created strains [25]. Cells carrying temperate P1 phage DNA as a large plasmid are also prevented from further P1 phage infection. This principle is used in cross-streak agar assays to detect the presence of temperate phages in the sample. In this technique, the colonies to be tested are streaked across a ‘P1 lysate layer’ on a LB agar plate, and the plate is incubated. If colony growth is not observed on the streak beyond the lysate layer, then it confirms the absence of temperate P1 phages from the sample. However, if growth is observed beyond the lysate layer, this indicates either the presence of temperate P1 phages or immunity of the strain to P1 phage infection (Fig. 2b) [25]. We used the cross-streak agar assay to test the colonies obtained from the phage transduction experiment as well as control strains (Methods section IID). We observed that 14 out of the 18 tested colonies from the phage transduction experiment grew across the P1 lysate streak, thereby demonstrating the presence of temperate bacteriophages in these transduced colonies (Fig. 2c). The remaining 4 colonies that were verified to be devoid of temperate phages can be used for further experiments.

The best practice to avoid contamination by temperate bacteriophages is to employ a verified virulent P1 lysate in P1 phage transduction experiments. To facilitate this verification of the P1 lysate, we have developed a rapid, easily applicable assay to detect the presence of temperate phages in the P1 lysate or in the employed strains. This assay is derived from Evans Blue-Uranine (EBU) plate assays, which are commonly used to verify pseudo-lysogeny in P22 phage transduction experiments of *Salmonella* strains [35]. We demonstrate here its first use in P1 phage transduction experiments using *E. coli* cells. When temperate phages are present in cells, a colony formed from these cells will have a pH that differs from that of uninfected cells as a result of pH lowering through the lysis of pseudo-lysogenic cells [36]. This property has been exploited in the EBU plate assay to directly visualize colonies containing Evans blue stained pseudo-lysogenic cells (Fig. 2d).

The temperate phage-infected cells verified by the cross-streak agar experiment were tested using the EBU plate assay (Methods section IIE): they exhibited exclusively dark green colonies (Fig. 2e), which we associate with infection by temperate phages that results in a change in cellular pH [36]. Conversely, the cells verified to be free of phages displayed exclusively pale green colonies (Fig. 2f). To demonstrate that even minute contamination of temperate phages in P1 lysate could be detected, we mixed the contaminated lysate with a verified virulent P1 stock of the *Ypet-DnaN* strain at a ratio of 1:100. We performed a P1 phage transduction experiment as described above, and inspected 20 random colonies using EBU plate assay. The results showed that 3 of the inspected 20 EBU plates contain few colonies which are dark green in color, whereas neighboring pale yellowish green-colored colonies are free of phages (Fig. 2g), confirming the detectability of low-level temperate phage contaminants in P1 lysate. We find that the EBU plate assay is more convenient and reliable than the cross-streak agar assay for the detection of temperate phage contamination in transduced colonies and P1 lysates, and the phage-free colonies can be used for further research.

### Evaluation of *E. coli* strains based on cellular growth or morphology characteristics

**Fig. 3:**
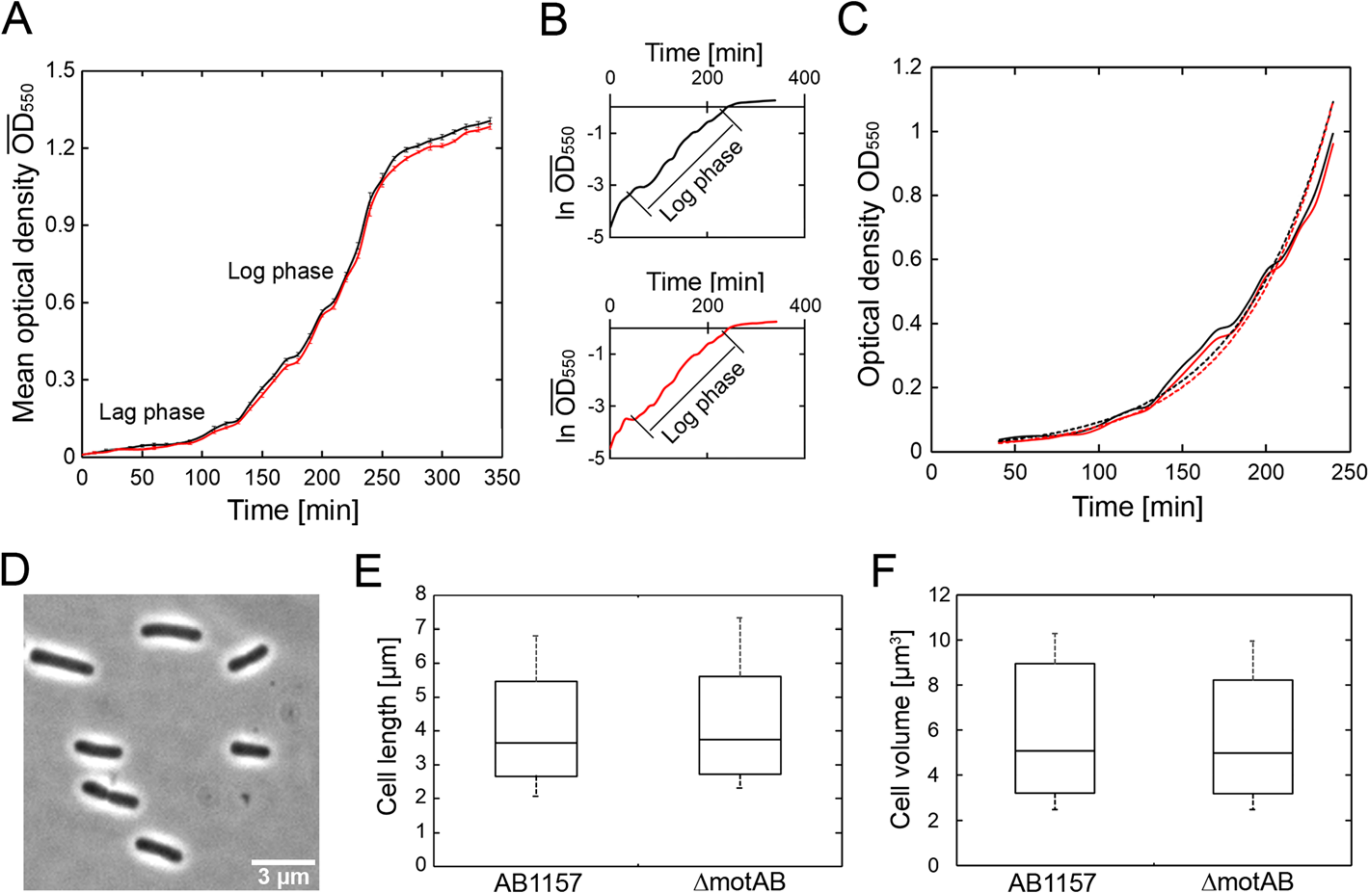
Evaluation of *E. coli* strains based on cellular growth or morphology characteristics. **a** Growth curves of the AB1157 (black) and recombineered *ΔmotAB* cells (red) in shake flasks containing LB medium at 37 °C and 250 rpm. **b** A simple method using semi-log plot to find the log phase of the growth curve for AB1157 (black) and *ΔmotAB* (red) strains. The linear region of the semilog plot is the log phase of the growth curve. **c** The exponential fitting of the selected log phase of the growth curve for AB1157 (black) and *ΔmotAB* (red) strains. From the fit (dotted lines), the growth rates (μ) are determined as 1.11 h^−1^ and 1.13 h^−1^ for one sample of AB1157 and *ΔmotAB* strains repectively. **d** A sample phase contrast image of AB1157 cells which were grown in LB medium at 37 °C and 250 rpm is shown. Such images were analyzed by MicrobeTracker software to calculate precisely the cell length and volume for each cell. **e & f** The data of cell length and cell volume of ~350 cells for each strain are plotted using a Box and Whiskers plot. The line within the box corresponds to the median value, the borders show the upper and lower quartiles (75 % and 25 %), and the whiskers represent the maximum and minimum values

Bacterial growth curve analysis provides an overview of the growth behavior of the chromosomally engineered *E. coli* strains. A typical bacterial growth curve starts with a lag phase as the bacteria adapt to the fresh growth medium, followed by a log phase in which growth is exponential. The final phase of the growth curve displays stationary growth as a result of nutrient scarcity, after which cells eventually die (Fig. 3a) [37]. Two important parameters that can be determined using the technique of growth curve analysis are the log-phase growth rate (μ) and the duration of lag phase (τ_l_) [38]. The log phase doubling time (generation time, τ_d_) is calculated from μ. If growth defects are introduced during the strain creation process, they can be detected by comparing the generation times of the parental strain with those of the created strain. The literature suggests numerous models and tools with which to perform this analysis [37, 38]. As an example, we have performed growth curve validation for the AB1157 and *ΔmotAB* strains (Fig. 3a, Methods section IIF). The critical step is to determine which time points of the growth curve fall in the log phase; fortunately, this is easily achieved by determining the linear region of the semi-log plot of the same curve (Fig. 3b). By fitting the log phase portion of the curve with an exponential function, we calculated the growth rates for each sample (Fig.3c). From the growth rates, the mean generation times with standard deviation (SD) for the AB1157 and *ΔmotAB* were found to be 39.2 ± 2.1 min and 38.7 ± 1.9 min, respectively. To determine the statistical significance of this difference, we employed *t*-test statistics for two independent sample means [39]. From the observed p-value of 0.68 (Table 4), we conclude with 95 % confidence intervals that no significant difference in generation times can be attributed to the *motAB* deletion genotype.

**Table 4:** “*t*-test” statistics for two independent samples of AB1157 and *ΔmotAB* strains’ generation time, cell volume and cell length

Parameter	Mean		Standard deviation (σ_Mean_)		*t*-value	*p*-value
Sample	AB1157	*ΔmotAB*	AB1157	*ΔmotAB*		
Generation time [min]	39.2	38.7	2.1	1.9	0.43	0.68
Cell volume [μm^3^]	5.5	5.3	1.8	1.6	1.55	0.12
Cell length [μm]	3.3	3.4	0.9	0.7	−1.64	0.1

Cell morphology can be examined using numerous methods including flow cytometry [40], atomic force microscopy [41], among others, and this essential phenotype can reveal the overall fitness of the chromosomally modified strain. Here we describe an approach that employs phase contrast microscopy and automated image analysis software MicrobeTracker (Methods section IIG) [42]. Using this open-source software, numerous indicators of cellular physiology such as cell volume and cell length can be determined simultaneously in an automated fashion from phase contrast images of cells (Fig. 3d). To illustrate this approach, we acquired images of AB1157 and *ΔmotAB* cells grown in LB medium at 37 °C, and for each strain we analyzed approximately 350 cells. The mean cell volume (with SD) of AB1157 was found to be 5.5 ± 1.8 μm^3^ while that of *ΔmotAB* was 5.3 ± 1.6 μm^3^. In the same analysis, the mean cell length for AB1157 strain was found to be 3.3 ± 0.9 μm (Fig. 3e), and that of *ΔmotAB* was 3.4 ± 0.7 μm (Fig. 3f). *t*-test statistics were used to determine any significant cell shape defects in the strains (Table 4) and revealed that the mean cell volume and cell length are not significantly different between the AB1157 and *ΔmotAB* strains using 95 % confidence intervals from the observed p-values (~0.1).

### Application of the validation methods in a strain engineered at a different chromosome locus

**Fig. 4:**
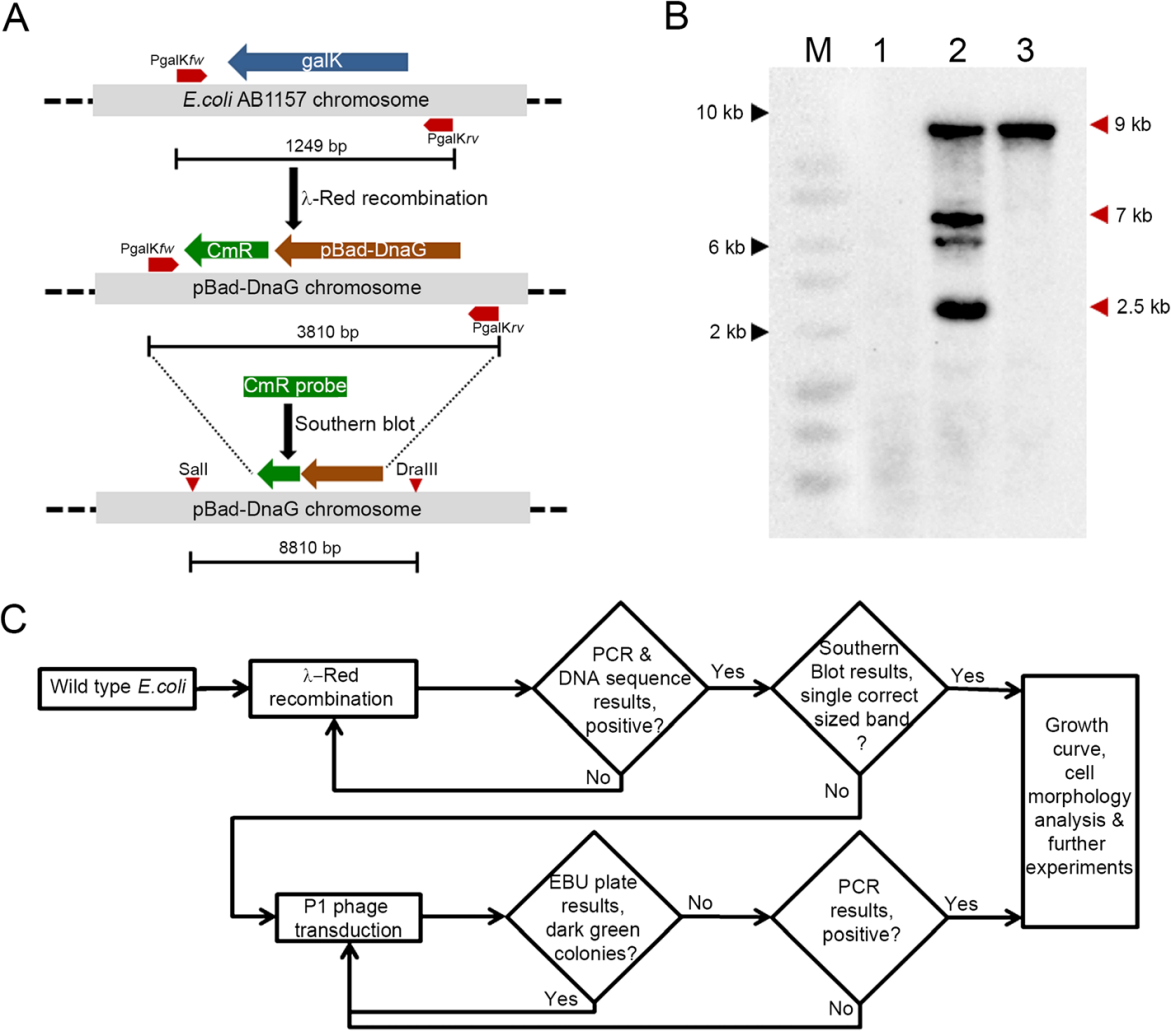
Application of the validation methods in a strain engineered at a different chromosome locus. **a** The scheme depicts the replacement of the endogenous non-essential *galK* gene with an arabinose inducible primase gene (*pBad-DnaG*) along with a *CmR* gene through λ-Red recombination. The positions of the flanking primers for the *galK* gene (PgalK*fw* and PgalK*rv*) are marked, and the corresponding product lengths from PCR are indicated, along with the probed region in chromosome and the expected band size from Southern blot analysis. **b** The Southern blot results for the different experiments. Lane 1: the AB1157 sample in lane 1 has no insert, as expected. Lane 2: the *pBad-DnaG* strain obtained through recombineering has multiple bands (mainly at ~9 kbp, 7 kbp, 6.5 kbp, and 2.5 kbp). Lane 3: the *pBad-DnaG* strain after P1 phage transduction of the intended locus into the wild type AB1157 strain displays one band (~9 kbp) at the right fragment size, showing that the extraneous insertions can be removed in the final strain using this approach. **c** A flow diagram summarizing the sequence of the various validation techniques that should be performed prior to subsequent usage of the chromosomally engineered *E.coli* strain

To demonstrate the effectiveness of the validation methods described here, we applied them to a strain that is chromosomally engineered at a different locus using λ-Red recombination. In this strain, we replaced the endogenous non-essential *galK* gene in the *E.coli* AB1157 chromosome with an arabinose inducible primase gene (*pBad-DnaG*) along with *CmR* gene (Fig. 4a). We performed Southern blotting and we observed from its results that extraneous insertions occurred in the created strain along with the intended insertion. We then applied P1 phage transduction using verified virulent P1 phages to transduce the intended insertion into the wild type *E.coli* AB1157 strain. Again using Southern blotting, we found that the P1 phage transduction step effectively produced the desired strain, fully devoid of extraneous insertions (Fig. 4b).

## Conclusions

We have consolidated and explained in detail simple, yet reliable, validation techniques which may be applied to verify chromosomally engineered *E. coli* strains for non-specific insertions in the chromosome, temperate phage contamination and general phenotype defects in growth and cell-shape. We have described the aspects of strain verification that common approaches such as PCR and DNA sequence analysis do not report on, such as the presence of extraneous insertions verified by Southern blot analysis after recombineering. Additionally, we have also adapted and described in detail the EBU plate assay for the validation of *E. coli* strains created by the commonly used approach for chromosomal insertion, P1 phage transduction. In comparison to the cross-streak agar assay, we find the EBU plate assay to be more convenient and reliable. The validation methods discussed here are of widespread utility and can be applied to any chromosome engineering technique. A summary of the suggested workflow for the various validation steps that should be performed prior to using an *E.coli* strain for further experiments is shown in Fig. 4c.

## Methods

All the chemicals and biological reagents used in this study were ordered from Life Technologies (Europe). The *E. coli* strains used in this research are the AB1157 strain [43] and its derivatives. Optical density measurements were performed using the cell density meter Ultraspec 10™ from GE Healthcare Europe GmBH (The Netherlands). For each of the techniques described in the main text, brief and specific descriptions are provided below, together with a detailed, step-wise protocol. Our adaptations or improvements to standardized protocols are indicated in italics.

### Strain creation methods

In this study we performed λ-Red recombination [17] and P1 phage transduction [31] for the strain creation using the following protocols.

#### λ-Red recombination (or recombineering)

This protocol is adapted from the literature [17, 24].

1. Perform PCR using a high fidelity polymerase (e.g. Phusion polymerase from New England Biolabs® Inc) from a *linear or linearized DNA template* containing an antibiotic resistance gene (e.g. chloramphenicol resistance (*CmR*)) by specially designed primers (~70 bp) for generating homologous regions at the ends.
2. *Gel-purify the PCR product.*
3. Transform a competent strain of interest with a temperature sensitive plasmid coding for λ phage’s Red recombinase proteins (e.g. pKD46 [17]) as described [44] and grow the cells overnight on a agar plate at 30 °C in the presence of the corresponding antibiotic.
4. Pick a colony from this plate and grow the cells overnight in *5 mL* LB medium containing respective antibiotics at 30 °C*, while shaking at 250 rpm.*
5. Prepare three samples of 1.4 mL LB medium with antibiotics, *50 μL of the overnight culture of cells containing the pKD46 plasmid, 0.2 % arabinose (or appropriate inducer used) along with one sample without arabinose in separate microfuge tubes.*
6. Incubate the cultures for *1.5 to 2 h at 37 °C*, shaking at *750 rpm*, with the lids punctured.
7. Centrifuge the samples for 30 s at 12,000× g.
8. Discard the supernatant, and place the samples on ice.
9. Re-suspend the pellet with 1 mL chilled and sterile double distilled water.
10. Repeat the centrifugation and re-suspend the pellet again in double distilled water *3 times* more.
11. Centrifuge the samples for 30 s at 12,000× g, and *remove the supernatant until ~50 μL is left in the tubes*.
12. Re-suspend the pellet in remaining volume, and keep it on ice.
13. *Add 1–3 μg of purified PCR product with a volume not more than 5 μL from step 2 to the samples on ice*.
14. *Include the following controls: a plasmid with same antibiotic selection marker as a positive control and sterile water as negative control*.
15. Pipette the entire volume from a sample to an electroporation cuvette, and electroporate the cells at 1250 Volts using an electroporator (Eppendorf®).
16. Re-suspend the cells in the cuvette with 1 mL of SOC medium *by pipetting up and down, and transfer them to a new sterile microfuge tube*.
17. Incubate the samples *in lid-punctured microfuge tubes for 70 min at 37 °C while shaking at 750 rpm, in order for the recombination to occur.*
18. Pour LB agar plates containing an appropriate concentration of the selection antibiotic. Do not add the antibiotics required for the temperature sensitive plasmid since the plasmid will be lost during the culture.
19. *Centrifuge the samples at 12,000× g for 30 s, and remove 900 μL of the supernatant.*
20. Re-suspend the cells in remaining volume, and plate them onto LB agar plates.
21. Incubate the plates overnight at 37 °C.
22. *Re-streak 10 colonies on a plate with the selection antibiotic, and incubate the plate overnight at 37 °C.*

#### P1 phage transduction

The protocol of P1 phage transduction that we adapted from the literature is explained here [31]. The protocol consists of two steps: (i) P1 lysate preparation from donor strain and (ii) Phage transduction to recipient strain. *However, the infectivity of the source P1 lysate stock should be determined first by using spot agar assay*(Methods section IIC)*.*

i. P1 Lysate preparation
  1. Inoculate the recipient strain and the donor strain from the LB agar plates (e.g. from Methods section IA step 22) in *5 mL of LB medium with respective antibiotics, and grow the cells overnight at 37 °C with shaking at 250 rpm.*
  2. *Dilute in duplicate 0.5 mL of the overnight donor culture into 4.5 mL of LB medium containing 60 μL of 1 M CaCl*_*2*_*and 120 μL of 1 M MgSO*_*4*_*.*
  3. Incubate the cultures at 37 °C for 45 min.
  4. *Add 100 μL of the P1 phage lysate stock that has been prepared by infecting the wild type E.coli strain. The volume of P1 lysate used may vary depending on its infectivity (For example, if the infectivity value of P1 lysate is 10*^*9*^*pfu mL*^*−1*^*, then use 100 μL lysate).*
  5. Continue the incubation until the culture is lysed or cell clumps are visible (usually around 3 to 4 h). The control culture without phages should show normal growth.
  6. *Add 4 to 5 drops of chloroform to the lysed culture, and stir the mixture well using a vortex mixer*.
  7. *After leaving the mixture to clarify for 5 min, transfer the upper liquid layer to a new sterile tube, and centrifuge the liquid for 20 min at 4200× g, 4 °C*.
  8. Pass the upper lysate layer further through a 0.45 μm filter to remove any viable donor cells.
  9. Store the P1 lysate devoid of chloroform at 4 °C for future use.
ii. Transduction
  1. Dilute 100 μL of the overnight recipient culture in 900 μL *LB medium containing 75 mM CaCl*_*2*_*and 150 mM MgSO*_*4*_*in 5 microfuge tubes*.
  2. *Add 5 μL, 50 μL, 100 μL or 200 μL of P1 lysate both to the 4 tubes and, as a control, to the one tube without lysate. The volumes of P1 lysate used may vary depending on its infectivity. (For example, if the infectivity value of P1 lysate is 10*^*9*^*pfu mL*^*−1*^*, then use the volumes mentioned here)*.
  3. Incubate the cultures at 37 °C *while shaking at 250 rpm for 30 min*. Infection of recipient cells occurs in this step.
  4. Centrifuge the cells at *12,000× g for 3 min*, and discard the supernatant.
  5. Re-suspend the pellet in 1 mL of LB medium containing 20 mM sodium citrate *(pH 5.5)* to reduce the infectivity of the adsorbed P1 phages by chelating the divalent ions. The transduction occurs during this step.
  6. Incubate the cells for *1.5 to 2 h at 37 °C with sufficient aeration and shaking at 250 rpm.*
  7. *Centrifuge the cells and discard the supernatant.*
  8. *Repeat step 6 and 7 twice to remove the phages as much as possible.*
  9. Re-suspend the pellet in *100 μL LB medium containing 20 mM sodium citrate (pH5.5)*.
  10. Plate the cultures onto LB agar plates containing 20 mM sodium citrate with respective antibiotics for selection.
  11. *Re-streak ~16 colonies onto LB agar plates containing 20 mM sodium citrate and selection antibiotics.*

### Strain verification methods

Here we describe the step-by-step methodology of the general validation techniques for chromosomal engineered *E. coli* strains.

#### PCR and DNA sequencing

For the high throughput verification of the individual colonies that are obtained from the strain creation methods described above, a simple analytical PCR and DNA sequencing are the widely used verification steps. The accompanying steps are described below.

1. *Design primers for PCR in such a way that the sequence flanks the region of interest in the chromosome, and the primer binding sites are not farther than 100 bases from the recombination site.*
2. *Re-suspend each colony to be verified by PCR in 50 μL of sterile water, and streak 10 μL on LB agar plates containing antibiotics.*
3. *Extract the DNA from the remaining cells into water by boiling the samples for 5 min and centrifuging them at 12,000× g for 1 min.*
4. Perform a PCR reaction on this DNA using the primers designed in step 1.
5. Verify the length of the PCR products using agarose gel electrophoresis, and select colonies with proper insert length for DNA sequence analysis of the corresponding PCR product using the same flanking primers. *If necessary, use specific internal sequencing primers to verify the correct insertion in the chromosome.*
6. Compare the DNA sequences by aligning it with the corresponding theoretical sequences to check for any point mutation or deletion introduced during the strain creation process.

#### Southern blotting

The copy number of the recombined DNA in the chromosome can be easily verified by using Southern blotting [34]. In this study, we used the AlkPhos® Direct labelling and detection system manufactured by Amersham™ (GE healthcare Europe GmbH, The Netherlands) because it is specially developed and well optimized for blotting experiments. DNA extraction was performed using Qiagen™ DNA isolation kit. We used high fidelity restriction enzymes manufactured by New England Biolabs® Inc.

1. Extract the genomic DNA from the strains to be verified. *Include the genomic DNA wild type strain as control.*
2. *Select two or three restriction enzymes using following criteria. The restriction sites must flank the region of interest and should not be contained within the region itself. Since it is difficult to resolve DNA fragments larger than 10 kb through gel electrophoresis and to achieve the best resolution, it is a good practice to make sure the size difference between the restricted fragment and the region of interest (usually < 2 kb) does not exceed 7 kb.*
3. Perform the restriction digestion of *~10* μ*g* genomic DNA samples *overnight preferably using the high fidelity restriction enzymes to avoid star activity* [45]*.*
4. Separate the digested DNA samples in a 0.8 % agarose gel by running electrophoresis *overnight at a constant current of 15 mA*.
5. Depurinate the DNA fragments by incubating the gel for 15 min in 0.1 M HCl solution on a plate shaker, and wash subsequently four times with double distilled water.
6. Denature the fragments in denaturing buffer (1.5 M NaCl and 0.5 M NaOH) on a plate shaker for 15 min, and wash four times with double distilled water.
7. Incubate the gel for 15 min in neutralizing buffer (1.5 M NaCl and 0.5 M Tris base, pH 7.5) while shaking, and wash four times with double distilled water.
8. Transfer the DNA fragments by capillary action to a pre-soaked Hybond-N+ membrane (GE Healthcare) using 20 × SSC buffer (3 M NaCl and 0.3 M tri-sodium citrate).
9. Pre-hybridize the blot with hybridization mix (35 mL AlkPhos® Direct hybridization buffer, 1 g NaCl and 1.4 g blocking agent-GE Healthcare) for *30 min at 55 °C in a rotary mixer*.
10. *Amplify the region of interest using specific PCR primers from the source DNA (usually a template plasmid or wild type chromosome). The optimal size of the PCR product is ~200 to 1000 bp.*
11. Denature the PCR product by boiling for 5 min, chill it on ice, and label it using appropriate reporters (e.g. *thermo-stable alkaline phosphatase*) that can catalyze non-luminescent substrates and yield luminescent products. The labeled PCR product can then be used as a DNA probe.
12. Add the DNA probe to the membrane in the hybridization buffer, and hybridize the probe in a rotary mixer at 55 °C overnight.
13. Wash the membrane in rotary shaker at 55 °C with 100 mL wash buffer 1 for *10 min* (pH: 7; 2 M Urea, 0.1 % SDS, 0.15 M NaCl, 0.05 M NaH_2_PO_4_, 1 mM MgCl_2_, and 1 g Blocking reagent)
14. Wash the membrane twice with 100 mL wash buffer 2 (pH: 10; 3 g Tris base, 2.8 g NaCl and 0.2 M MgCl_2_) in plate shaker at room temperature for 10 min.
15. Incubate the membrane, with 3 mL non-luminescent substrate for *5 min* and dry the membrane.
16. Wrap the membrane using Saran™ wrap, and detect chemi-luminescence on the blot using appropriate detectors. *The resulting number of bands obtained is indicative for the copy number of the recombined DNA in the chromosome.*

#### Spot agar assay

The infectivity of the phages in a P1 lysate can be determined by a spot agar assay. This method is adapted from Ref. [31].

1. Add CaCl_2_ to the overnight culture of the recipient strain from the phage transduction step such that the final concentration is 5 mM.
2. Serially dilute the P1 lysate to the order of 10^−10^ using LB medium containing *75 mM CaCl*_*2*_*and 150 mM MgSO*_*4*._*Make sure to change the pipette tips during the dilution step.*
3. Mix 0.25 mL of cell culture with 2.5 mL of molten LB top agar (0.75 %) containing 2.5 mM CaCl_2_.
4. Pour the mixture onto LB agar (1.5 %) plates containing 2.5 mM CaCl_2_ and let it solidify.
5. Spot 10 μL of each phage stock dilution onto the lawn of cells.
6. Keep the plates upright, and after the spots are dry, incubate them at 37 °C overnight.
7. Calculate the titer value in pfu mL^−1^ by counting the number of plaques in the lowest concentration spot, then multiplying it with the order of dilution (e.g. 10^7^) and finally by the factor 100 (to account for 10 μL volume used).

#### Cross-streak agar assay

The presence of temperate phages in phage-transduced colonies can be tested by a cross-streak agar assay. This method is adapted from Ref. [25].

1. Prepare a LB agar plate containing *2.5 mM CaCl*_*2*_.
2. Draw a straight line on the back of plate across the middle and well-spaced dots on one side (Fig. 2b).
3. Holding the plate in a slanting position, pour *50* μ*L* of phage lysate on the LB agar from one end of the straight line.
4. Tilt the plate back to uniformly distribute the lysate around the straight line.
5. Take a colony or liquid overnight culture to be tested for temperate phages with the broad side of the inoculation needle.
6. Place a dot of the colony or culture at the marked location and leaving a few millimeters space streak the colony or culture perpendicularly across the phage lysate.
7. Transfer the plate carefully to incubator, and incubate overnight at 37 °C.

#### Evans Blue-Uranine (EBU) plate assay

The phage-transduced colonies can be tested for temperate phage contamination and can be cured of phages using EBU plate assay described below. The method is adapted from Ref. [46] for *E. coli* strains.

1. Make agar plates from 1 L of molten LB agar containing 40 mL of 12.5 % K_2_HPO_4_, 1.25 mL of 1 % Evans Blue stain solution, 250 μL of 10 % uranine solution and if necessary antibiotics.
2. *Store the plates in dark at 4 °C. Since Evans Blue stain is carcinogenic the plates must be handled carefully with gloves, and it is usually a good practice to autoclave and dispose in the carcinogenic waste.*
3. *Inoculate the colonies obtained from a phage transduction experiment in LB medium, and grow them for 3 h at 37 °C with shaking at 250 rpm.*
4. *Dilute the exponentially growing LB cultures 100 times, and spread 50 μL of the culture uniformly across the EBU plates.*
5. *Incubate overnight at 37 °C in the dark.*
6. *Analyze the colonies. The colonies without temperate phages appear pale yellow in color while those with temperate phages are colored dark green.*

#### Growth curve analysis

Growth curve analysis of the *E. coli* strains can be performed using shake flasks as described below. In this study, we used MS Excel and MATLAB to analyze the data.

1. *Dilute in triplicate overnight cultures of the wild type and the strain to be verified to an OD*_*550nm*_*of 0.01 in 50 mL fresh medium.*
2. Grow the cells at 37 °C in a shaking incubator (*250 rpm*).
3. *Use sterile disposable cuvettes and aseptic conditions to note the OD*_*550nm*_*of 1 mL aliquot from each culture sample using a cell-density meter.*
4. *Add back the culture aliquot to the sample after the measurement in order to keep the culture volume constant.*
5. Steps 3 and 4 are repeated at an interval of *15 min* until the OD_550nm_ is a constant value.
6. Plot the OD_550nm_ values against time in min to get a sigmoidal curve in this plot.
7. Plot the OD_550nm_ values in logarithmic scale, the exponential phase of the growth curve can be identified as the distinct linear part (Fig. 3b,c), and perform an exponential fit only on the OD_550nm_ values of this phase in the growth curve for each sample to find the growth rate (μ) [38].
8. Calculate the generation time (τ_d_) from μ using the formula: τ_d_ = *ln* (2)/μ.
9. *To test the reproducibility of results, repeat the experiment, and average the generation times for 6 samples of the strain to be verified. The result can then be compared to the average generation time of the wild type strain using t-test for two independent sample means* [39]*.*

#### Detection for cell-shape defects by microscopy

Phase-contrast microscopy is a powerful technique to determine the cell-shape characteristics of a bacterium. In this study, it was performed using a Nikon Ti™ microscope with a 100× objective and an Andor iXon™ Ultra 897 EMCCD camera.

1. *Grow the LB culture of the strain to be verified until it reaches the exponential phase OD*_*550nm*_*is ~ 0.3 to 0.4.*
2. *Centrifuge 1 mL of the culture at 12,000× g for 1 min, and remove the supernatant.*
3. *Re-suspend in 250 μL of fresh medium, and pipette 5 μL of concentrated culture on a pad of agarose (1.5 %) flattened on a microscopic slide. Let the fluid evaporate.*
4. *Place a sterile and clean cover-glass on top of the cells, and press gently using tweezers.*
5. *Analyze the cells on a phase contrast microscope using an objective with a magnification >60× and a digital camera.*
6. *For a robust analysis, acquire the images with at least ~100 separate cells within a field of view.*
7. Store the images in greyscale tiff format, and analyze them using MicrobeTracker software to accurately determine the cell shape defects [42].
8. Export the analyzed data from the software in CSV format for further analysis e.g. *t-test statistics* and representation of data, e.g. *a Box and Whiskers plot.*
